# CD49b/CD69-Dependent Generation of Resting T Helper Cell Memory

**DOI:** 10.3389/fimmu.2013.00183

**Published:** 2013-07-10

**Authors:** Asami Hanazawa, Max Löhning, Andreas Radbruch, Koji Tokoyoda

**Affiliations:** ^1^Deutsches Rheuma-Forschungszentrum (DRFZ), Berlin, Germany; ^2^Department of Rheumatology and Clinical Immunology, Charité – University Medicine Berlin, Berlin, Germany

**Keywords:** T helper cells, immunological memory, bone marrow, CD49b, CD69, homing

## Abstract

In the absence of antigen, memory T helper (Th) cells are maintained in a resting state. Recently it has been shown that bone marrow (BM) is a major reservoir of resting memory Th cells. In a given immune response, less than 10% of the activated CD4 T cells are recruited to the pool of resting BM memory Th cells. Here we review recent evidence that CD69 and CD49b control homing of memory Th cell precursors to the BM. During the effector phase of an immune response, about 10% of activated CD4 T cells in the spleen express both CD69 and CD49b, and thus qualify as precursors of resting memory Th cells of BM. Loss or blockade of CD69 and CD49b expression on CD4 T cells impairs the generation of resting memory Th cells in the BM. Moreover, in the absence of BM memory Th cells in CD69-deficient mice, T-cell help for B cells is impaired, confirming the central role of BM memory Th cells in the maintenance of immunological memory.

## Introduction

The immune system can memorize previously encountered specific antigens for many years, both by the secretion of specific antibodies (humoral memory) and by long-lived, pre-activated cells (reactive memory). It is still debated whether immunological memory is dependent on persistent antigen (1, 2) and whether it is dependent on long-lived effector cells as opposed to dedicated memory cells (3). Here we define immunological memory as “the maintenance of information in the absence of the original instruction”, i.e., memory cells are maintained in the absence of antigen (4). This definition distinguishes “resting” long-lived memory cells from the long-lived effector cells that develop during infection and persistent immune responses. Recent evidence indeed points to the existence of distinct professional memory lymphocytes. In particular, long-lived “memory” plasma cells have been identified, which mostly reside in the bone marrow (BM) and provide humoral memory (5–7). Recently, also professional memory T helper (Th) cells have been located in the BM. They represent up to 10% of the Th cells generated in an immune reaction. It has been shown that they rest in terms of proliferation and gene expression (8). This finding provides a fascinating challenge for the understanding of how these memory Th cells are generated, maintained, and reactivated. It also raises questions about their relation to long-lived effector Th cells out of the BM. Here we review recent data about roles of CD49b and CD69 in generation of the precursors of memory Th cells and discuss new concepts on how BM memory Th cells are generated and on the particular role of the BM in this process.

## Resting Memory

**Figure 1:**
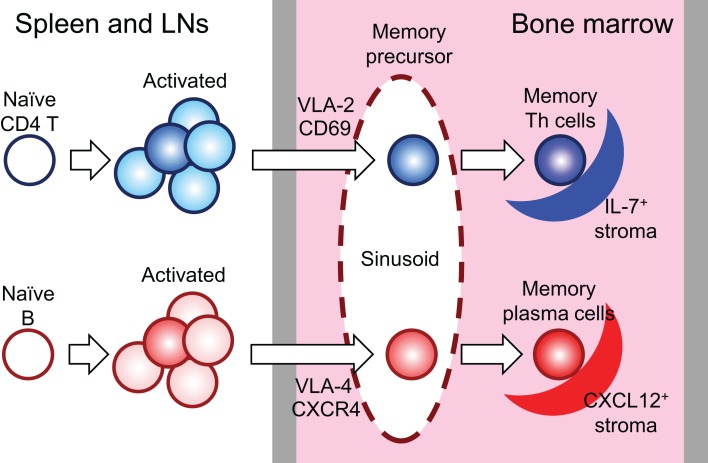
**Generation, relocation, and maintenance of memory Th and plasma cells**. In an immune response, 10–20% of activated lymphocytes in secondary lymphoid organs relocate into the BM and reside and rest there in the distinct stromal niches. LNs: lymph nodes.

Memory Th cells, memory B cells and memory plasma cells are in a resting state, i.e., they rest in terms of proliferation (5, 7, 9, 10), while for memory CD8 T cells this is likely, but has not been formally demonstrated so far. Memory B cells are thought to be generated in the germinal centers (GCs), although some studies report that early memory B cells develop outside of GCs (11–13) and that affinity-matured IgM^+^ memory B cells develop in GCs (14, 15). Memory plasma cells are generated from activated B cells, in a way not fully understood, alternative to memory B cell differentiation (16, 17). While it has been described that memory B cells are maintained in the spleen (18, 19), memory plasma cells are maintained mostly in the BM (5, 6). Memory plasma cells of the BM are maintained in distinct survival niches, organized by CXCL12-expressing reticular stromal cells, but also containing other cells, like eosinophils, providing essential survival factors (10, 20, 21). A survival niche for memory B cells has not been identified so far. Memory plasma cells themselves are immobile. Although they express CXCR4, they do not migrate in response to its ligand CXCL12, while their immediate precursors, the plasmablasts do (22). Thus, it seems reasonable to assume that plasmablasts immigrate into the BM, dock onto CXCL12-expressing stromal cells and there differentiate into memory plasma cells [Figure 1; (7, 23)].

## Numerical Control of Resting Th Cell Memory in BM

More than 95% of antigen-specific memory Th cells in the BM individually colocalize with VCAM-1^+^IL-7^+^ stromal cells, which comprise about 2% of the total BM cell populations (8). These IL-7-expressing stromal cells probably form a survival niche for resting memory Th cells within the BM. Studies show the competition of newly generated memory Th cells with older ones (24, 25), suggesting the number of niches may be limited; this may determine the overall capacity of the BM for memory Th cells. We compared immune responses to a lymphocytic choriomeningitis virus (LCMV) peptide with responses to a natural LCMV infection and found that they differed substantially in terms of magnitude; these results provide insight as to how a limited number of BM survival niches regulate the size of the memory T-cell pool. In the memory phase of each immune response, the absolute numbers of specific memory Th cells in the BM during each of the responses were comparable (unpublished data). In addition, we detected similar absolute numbers of antigen-specific memory Th cells in the BM in responses to ovalbumin (OVA) plus lipopolysaccharide (LPS) or in response to OVA plus Alum; however, the numbers of effector cells specific for these antigens were different. These numbers games suggest that the BM efficiently restricts access by activated CD4 T cells derived from the pool of memory Th cells via a mechanism that probably involves IL-7-expressing stromal cell, which organizes a niche for exactly one memory Th cell [Figure 1; (23)].

The maintenance of Th cell memory is dependent on IL-7, because long-lived CD4 T cells isolated from the spleen and lymph nodes and then adoptively transferred into IL-7-deficient mice do not survive (26). Persistence of antigen at low levels, i.e., TCR signaling, has been suggested to be required to maintain Th cell memory (1, 2). However, studies using MHC class II-deficient mice (27) and targeted deletion of the TCR (28) show that the maintenance of Th cell memory is independent of TCR-mediated signaling. Professional memory Th cells of BM dock onto IL-7-expressing reticular stromal cells, and there are no MHC class II-expressing cells in their immediate neighborhood (8). Thus it seems likely that the IL-7-expressing stromal cells organize survival niches for memory Th cells of the BM and that TCR stimulation is not required for the maintenance of resting memory Th cells of BM (23). If it is so important for the memory Th cell to be in this dedicated BM niche, central questions for the understanding of immunological memory are: When and how do the T cells get there, and which T cells do get there?

To determine the dynamics of the establishment of professional memory Th cells in the BM, we tracked murine antigen-specific CD4 T cells, generated in intentional immune responses to a defined antigen, into the memory phase of immune responses (8). In immune responses of naive TCR-transgenic CD4 T cells, specific for LCMV or OVA, or wild-type CD4 T cells in response to 4-hydroxy-3-nitrophenylacetyl keyhole limpet hemocyanin (NP-KLH), antigen-specific CD44^hi^ CD4 T cells were present in spleen and lymph nodes on day 4 and 7 (8). Then, the numbers of CD44^hi^ T cells in the secondary lymphoid organs decreased; however, there was an increase in the number of antigen-specific CD44^hi^ CD4 T cells in the BM. From day 30 to 60 post-immunization, more than 80% of the antigen-specific CD4 T cells were in the BM. In the BM they persisted in constant numbers for the rest of the observation period, 7–20 weeks. Beyond day 30 the antigen-specific Th cells are maintained in a resting state in the BM; these cells are not proliferating, having switched off most of their genes. When exactly the cells differentiate from activated cells into resting cells remains to be determined, as well as whether the T cells in BM also rest in terms of mobility.

## CD49b/CD69-Dependent Generation of Resting Th Cell Memory

The phenotype of resting memory Th cells of BM is clearly distinct from that of long-lived Th cells in the secondary lymphoid organs and in the blood. They share high expression of CD44, and low expression of CD62L. But only the memory Th cells of BM highly express Ly-6C. They also express CD49b and CD69. While the role of Ly-6C is still enigmatic, it should be noted that humans do not have an orthologous gene. It could be speculated that activated, Ly-6C^hi^ CD4 T cells of the secondary lymphoid organs are precursors of BM memory cells. But whether and if so, how Ly-6C is involved in their homing into the BM or their local maintenance remains unclear.

CD49b, or alpha2 integrin, together with beta1 integrin forms an adhesion molecule VLA-2, which binds mainly to collagens I, II, and XI (29, 30). BM-resident memory Th cells express both, VLA-2 and VLA-4, the alpha2- and the alpha4- beta1-integrin heterodimers. Notably, VLA-4 is expressed also by memory plasma cells, and antibodies to VLA-4 have been shown to eliminate plasma cells from BM (31). For memory Th cells this has not been tested so far. However, the homing of adoptively transferred BM memory Th cells to BM can be efficiently blocked by antibodies to alpha2 integrin (8). This observation alone points to a decisive role of CD49b in the trafficking of memory Th cell precursors to the BM niches for memory Th cells. It should be noted that among the putative ligands of VLA-2 are the collagens II and XI, and collagen XI is exclusively expressed in the BM (32). However, how collagen XI might guide memory Th cell precursors to their desired location remains to be determined.

CD69 is a member of the C-type lectin family and its extracellular ligand is unknown. It is expressed constitutively by more than 60% of the resting memory Th cells of BM (8, 33). This is surprising in the first place, since in general CD69 is considered as an activation marker, expressed by T cells activated in secondary lymphoid organs (34, 35). Jason G. Cyster and his colleagues then showed that CD69 in secondary lymphoid organs essentially acts as a retention signal (36). At first glance, retaining resting memory Th cells in the BM at times when their antigen is not around, would be a reasonable explanation for their expression of CD69. However, things seem to be more complicated. First of all, CD69-deficient mice do not develop a population of BM-resident memory Th cells, in the aftermath of an immune reaction (33). In the effector phase of an immune response, wild-type and CD69 ^−/−^ antigen-specific CD4 T cells can be detected readily, in comparable numbers in the secondary lymphoid organs and in the blood. Most notably, follicular helper T cells and GC B cells are present in numbers and phenotypes undistinguishable between wild-type and CD69^−/−^ mice. However, transition of CD69^−/−^ memory Th cell precursors into the BM is impaired. Very few CD69^−/−^ CD4 T cells arrive in the BM and those that do, do not colocalize with IL-7-expressing stromal cells (33).

## Differentiation into Resting Memory Cell Precursors

In mice, 10–20% of newly generated plasma cells persist in the BM during a secondary immune response (5, 7, 37, 38). In humans that have been vaccinated against tetanus, the proportion of specific antibodies that persists in the memory phase of the immune response is about 10% of that of the peak response, probably reflecting a corresponding elimination of 90% of the antibody-secreting cells had been generated (39). However, it is unclear how the 10% of memory plasma cell precursors, i.e., plasmablasts that finally make it are selected. In analogy to memory plasma cells, memory Th cells are generated from activated CD4 T cells, i.e., T-cell blasts. Upon immunization with protein antigens, about 10% of the splenic activated CD4 T cells generated at the peak of the response actually mature into BM memory Th cells. Does this mean that 10% of the T-cell blasts make it into the BM memory pool? And how are they qualified? We showed that both CD49b and CD69 are essential for the homing of memory Th cell precursors to the BM (8, 33). On day 6 post-immunization, about 10% of activated CD4 T cells expressed both CD49b and CD69 (unpublished data). It is an intriguing speculation, and hypothesis worthwhile testing, that coexpression of CD69 and CD49b by individual activated Th cells defines these cells as the precursors of resting memory Th cells. In addition, to improve vaccine efficacy, various vaccination strategies should be checked based on the efficiency in inducing CD69 and CD49b coexpressing memory Th cell precursors. Human BM CD4 T cells also preferentially express CD69 compared to peripheral blood CD4 T cells (40). The concept that memory Th cells reside and rest in the BM as observed in mice, may also extend to human Th cell memory and the particular role of the BM in memory maintenance, which should be investigated further.

## Conflict of Interest Statement

The authors declare that the research was conducted in the absence of any commercial or financial relationships that could be construed as a potential conflict of interest.
